# Access to Primary Care and Visits to Emergency Departments in England: A Cross-Sectional, Population-Based Study

**DOI:** 10.1371/journal.pone.0066699

**Published:** 2013-06-12

**Authors:** Thomas E. Cowling, Elizabeth V. Cecil, Michael A. Soljak, John Tayu Lee, Christopher Millett, Azeem Majeed, Robert M. Wachter, Matthew J. Harris

**Affiliations:** 1 Department of Primary Care and Public Health, Imperial College London, London, United Kingdom; 2 Division of Hospital Medicine, University of California San Francisco, San Francisco, California, United States of America; The University of York, United Kingdom

## Abstract

**Background:**

The number of visits to hospital emergency departments (EDs) in England has increased by 20% since 2007-08, placing unsustainable pressure on the National Health Service (NHS). Some patients attend EDs because they are unable to access primary care services. This study examined the association between access to primary care and ED visits in England.

**Methods:**

A cross-sectional, population-based analysis of patients registered with 7,856 general practices in England was conducted, for the time period April 2010 to March 2011. The outcome measure was the number of self-referred discharged ED visits by the registered population of a general practice. The predictor variables were measures of patient-reported access to general practice services; these were entered into a negative binomial regression model with variables to control for the characteristics of patient populations, supply of general practitioners and travel times to health services.

**Main Result and Conclusion:**

General practices providing more timely access to primary care had fewer self-referred discharged ED visits per registered patient (for the most accessible quintile of practices, RR = 0.898; P<0.001). Policy makers should consider improving timely access to primary care when developing plans to reduce ED utilisation.

## Introduction

The utilisation of emergency departments (EDs) is rising in several high-income countries. In England’s National Health Service (NHS), the annual number of hospital ED visits increased by 20% between 2007-08 and 2011-12 [1]. While in the U.S., where EDs increasingly act as a safety net for underserved patients, the annual number of ED visits increased by 23% between 1997 and 2007 [2]. The trends are unsustainable.

Some patients seen in EDs in England attribute their visit to the inability to see a primary care physician (general practitioner; GP) [3], lending support to the hypothesis that ED utilisation could be reduced by improving access to primary care. The Quality and Outcomes Framework, the U.K.’s primary care pay for performance programme, financially rewards general practices for reviewing patient access and its possible effect on ED utilisation [4]. Yet, this effect has not been empirically tested at a national level, and the evidence at a local level is inconclusive [5], [6].

This study examined whether more accessible general practices in England have fewer ED visits per registered patient. We focused on ED visits by patients whom a GP could have potentially managed or, at least, seen before the ED visit.

In contrast to studies conducted in the U.S., this hypothesis could be tested without the possibility of confounding by insurance status, due to the universal coverage of health services in England. In addition, as the NHS is a single payer system with a unified hospital database, the study could be conducted in a population of 54 million patients, making it the largest study of its kind to date.

## Methods

### Study Design and Setting

The study used a cross-sectional, population-based design with the general practice as the unit of analysis. The time period of data was 1^st^ April 2010 to 31^st^ March 2011. We included 7,856 general practices in the analysis, with a total registered population of 54,225,700, accounting for around 95% of practices in England [7]. The excluded practices had incomplete data for one or more variables, but had similar registered populations to the included practices in terms of age, sex, and ethnicity.

### Outcome Variable

The outcome variable was the number of visits at type 1 EDs (consultant-led 24 hour services with full resuscitation facilities) recorded as a self-referral and as discharged, either with follow-up treatment to be provided by a GP or without a requirement for follow-up treatment [5]. Those referred from other sources, such as a GP or the emergency services, and those that resulted in admission, transfer or referral to another healthcare provider, or death were excluded. Data were obtained from NHS Comparators [8], which displays aggregate ED data derived from the Hospital Episode Statistics ED minimum dataset.

### Measures of Primary Care Access

The predictor variables were measures of patient-reported access to general practice, obtained from the GP Patient Survey [9]. This annual survey, administered on behalf of the U.K. Department of Health, invites a sample of adults registered with a general practice in England to complete a validated questionnaire [10] regarding their experiences of and satisfaction with their practice; in 2010-11, 2.0 million patients completed a form. Individual responses are aggregated to the level of the general practice and weighted by age and sex to ensure representativeness of each practice’s registered population [11], [12].

The candidate measures of access for inclusion in the final model were the percentage of a practice’s registered population that, on their last attempt in the past six months, was able to see a GP within two weekdays; was able to book a GP appointment more than two weekdays in advance; had found it very or fairly easy to speak to a GP on the phone; and the percentage that see their preferred GP always, almost always or a lot of the time when an appointment is obtained. Patients were instructed to answer the questions associated with the first two of these variables only if such an attempt had been made. Practices were categorised into quintiles for each of these variables so that the relative difference in the outcome variable between the least and most accessible groups of practices could be observed.

Two additional variables from the GP Patient Survey were candidates to control for GP appointment demand: the percentage of the registered population that, in the past six months, had tried to see a GP within two weekdays; and the percentage that had tried to book a GP appointment more than two weekdays in advance.

### Control Variables

The analysis controlled for the age, sex, ethnic, socioeconomic, health and urban/rural profiles of each general practice’s population, in addition to the supply of GPs and relative travel time to the nearest hospital.

The percentage of a general practice’s registered population aged 65 years or over and the percentage that was male were calculated from data accessed via the NHS Information Centre Indicator Portal [7]. The practice percentage of white ethnicity was derived from Hospital Episode Statistics data using an externally validated method [13].

An Index of Multiple Deprivation (IMD) score, a measure of socioeconomic status, for each general practice was obtained from the NHS Information Centre Indicator Portal [7]. The practices were, first, ranked by their score and, then, categorised into quintiles with the least deprived practices forming the 1^st^ quintile and the most deprived forming the 5^th^ quintile. This adjustment was necessary as the IMD score does not characterise deprivation on a linear scale.

We used the prevalence of asthma, hypertension and obesity in each general practice’s registered population, as reported for the Quality and Outcomes Framework, to control for population levels of health [7]. The prevalence of hypertension had a moderate to strong positive correlation with that of other conditions, including coronary heart disease (*r* = 0.74), heart failure (*r* = 0.56), and stroke (*r* = 0.66); to reduce multicollinearity, we did not also include these conditions in the final model.

The urban/rural classification of a general practice’s location and the number of GPs per 1,000 registered patients were also obtained from the NHS Information Centre Indicator Portal [7]; a location was considered rural if its population was less than 10,000.

Data from the Department for Transport [14], [15] were used to calculate a registered population’s average travel time to the nearest hospital relative to that to the nearest GP by public transport and/or walking. The variable was defined in this way as a patient’s decision to see a GP or attend an ED is likely to be influenced by the relative difference in travel times, rather than the individual times alone.

Finally, indicator variables for the Strategic Health Authority in which a general practice is located were included to account for unobserved variation in regional health system characteristics and policy. The data obtained from the various sources were linked using the Organisation Data Service codes assigned to each general practice by the NHS.

### Statistical Methods

Negative binomial regression was used to test for an association between the outcome variable and the predictor and control variables. This was a suitable count model to use as the number of self-referred discharged ED visits was overdispersed. The natural logarithm of the general practice population size was used as an offset variable; its coefficient was constrained to unity so that the coefficients of the predictor and control variables could be interpreted in terms of an effect on the number of self-referred discharged ED visits per registered patient, referred to here as the rate of self-referred discharged ED visits.

The control variables, given above, were first entered into the model. The measures of patient-reported access were then entered and removed iteratively; those to be included in the final model were determined through observation of their statistical significance and minimisation of Akaike’s Information Criterion to assess model fit [16]. One measure of access was retained for inclusion: the percentage of the registered population that was able to see a GP within two weekdays. In order to control for the associated demand, the percentage that had tried to see a GP within two weekdays, irrespective of whether they were or were not then able to see a GP, was also included in the final model.

The effect sizes of associations are reported as rate ratios (RRs). For categorical variables, the RR can be interpreted as a 100(RR-1)% increase in the rate of ED visits relative to the rate for the reference group. For continuous variables, a one unit increase in their value is associated with a 100(RR-1)% increase in the rate of visits. All continuous variables were checked for non-linear relationships with the outcome variable. The variance inflation factor (VIF), a measure of multicollinearity, was less than five for all predictor and control variables, indicating that the assumption of no correlation among them was reasonably met. Possible interactions between predictor and control variables were also examined in exploratory analyses.

The null hypothesis stated that the measures of patient-reported access to general practice services would not possess a statistically significant association with the rate of self-referred discharged ED visits. An association with a *P*-value less than 0.05 was regarded as statistically significant. Analysis was conducted in Stata SE Version 12.1 (StataCorp, College Station, TX, USA).

## Results

Patients registered to the 7,856 included general practices made 4,537,622 self-referred discharged ED visits in England between April 2010 and March 2011 (Table 1). This accounts for 39.3% of all visits (11,538,268) and 61.3% of self-referred visits (7,402,722) to EDs by patients registered to these practices.

**Table 1:** Descriptive statistics for self-referred discharged ED visits, and characteristics of general practices and their registered populations in England, 2010-11.

Variable	Median	IQR*	Min.	Max.
**Number of self-referred discharged ED visits**	452.0	231.0 – 788.0	2.0	7508.0
**Rate of self-referred discharged ED visits** †	0.08	0.05 – 0.12	0.00	0.35
**Registered population size**	6084.5	3624.0 – 9347.5	762.0	40327.0
**Aged 65 years or over (%)**	15.8	11.8 – 19.1	0.0	45.3
**Male (%)**	49.8	48.9 – 51.1	39.0	76.1
**White (%)**	84.9	69.5 – 91.0	0.3	100.0
**Index of Multiple Deprivation (IMD)**	21.6	13.6 – 31.9	2.9	68.5
**Asthma prevalence (%)**	5.9	5.1 – 6.7	0.0	20.0
**Hypertension prevalence (%)**	13.8	11.7 – 15.9	0.0	37.4
**Obesity prevalence (%)**	10.7	8.3 – 13.3	0.0	32.3
**Number of GPs per 1,000 registered patients**	0.6	0.5 – 0.8	0.1	5.4
**Travel time to the nearest hospital relative to the nearest GP by public transport and/or walking**	2.5	1.9 – 3.4	0.8	13.0
**Had tried to see a GP within two weekdays (%)**	59.3	54.9 – 63.6	38.6	84.2
**Was able to see a GP within two weekdays (%)** ‡	82.0	74.0 – 89.3	25.0	100.0
**Had tried to book a GP appointment more than two weekdays in advance (%)**	46.6	41.0 – 51.5	6.1	77.1
**Was able to book a GP appointment more than two weekdays in advance (%)**	75.9	64.0 – 85.7	0.0	100.0
**Had found it very or fairly easy to speak to a GP on the phone (%)**	55.2	40.6 – 68.9	3.0	100.0
**See their preferred GP always, almost always or a lot of the time (%)**	73.4	61.9 – 83.3	14.3	100.0

7,856 general practices were included in the analysis.

Urban/Rural classification: Urban (n = 6,631); Rural (n = 1,225).

Strategic Health Authority: North East (n = 379); North West (n = 1,202); Yorkshire and the Humber (n = 748); East Midlands (n = 604); West Midlands (n = 916); East of England (n = 764); London (n = 1,424) South East Coast (n = 616); South Central (n = 492); South West (n = 711).

* IQR: interquartile range.

† Number of self-referred discharged ED visits per registered patient.

‡ The question in the GP Patient Survey associated with this variable was only completed by patients who had tried to see a GP within two weekdays in the past six months.

The median percentage of a practice’s registered population that had tried to see a GP within two weekdays in the past six months was 59.3% (IQR: 54.9–63.6%). This demand was not always met: the median percentage that was subsequently able to do so was 82.0% (IQR: 74.0–89.3%).

In the multivariable analysis, the percentage of the registered population that was able to see a GP within two weekdays had a statistically significant negative association with the rate of self-referred discharged ED visits (Table 2). Relative to the practices in the first quintile of this access variable, those in the second to fifth quintiles, providing more timely access to care, had fewer ED visits per registered patient. The model predicts a 10.2% (RR = 0.898; *P*<0.001) lower rate of visits for those practices in the fifth quintile relative to those in the first quintile.

**Table 2:** Multivariable regression model of the association between the rate of self-referred discharged ED visits and characteristics of general practices and their registered populations in England, 2010-11.

Variable		RR	*P*-value	95% CI
**Aged 65 years or over (%)**		0.989	<0.001	0.984 – 0.994
**Male (%)**		1.006	0.120	0.998 – 1.013
**White (%)**		1.000	0.489	0.999 – 1.001
**Index of Multiple Deprivation (IMD)**	**2.86 – 12.21**	–	–	–
	**12.22 – 18.10**	1.043	0.082	0.995 – 1.094
	**18.11 – 25.29**	1.186	<0.001	1.127 – 1.248
	**25.30 – 34.20**	1.270	<0.001	1.200 – 1.343
	**34.21 – 68.47**	1.417	<0.001	1.330 – 1.509
**Prevalence (%)**	**Asthma**	1.003	0.670	0.990 – 1.016
	**Hypertension**	1.002	0.615	0.994 – 1.009
	**Obesity**	1.006	0.021	1.001 – 1.011
**Urban/Rural classification**	**Urban**	–	–	–
	**Rural**	0.850	<0.001	0.811 – 0.890
**Number of GPs per 1,000 registered patients**		0.964	0.182	0.913 – 1.017
**Travel time to the nearest hospital relative to the nearest GP by public transport and/or walking**		0.974	<0.001	0.963 – 0.984
**Had tried to see a GP within two weekdays (%)**		1.007	<0.001	1.004 – 1.009
**Was able to see a GP within two weekdays (%)** *	**25.00 – 71.88**	–	–	–
	**71.89 – 79.23**	0.945	0.018	0.902 – 0.990
	**79.24 – 85.00**	0.926	0.002	0.883 – 0.971
	**85.01 – 91.11**	0.923	0.001	0.879 – 0.969
	**91.12 – 100.00**	0.898	<0.001	0.853 – 0.945
**Strategic Health Authority**	**North East**	–	–	–
	**North West**	0.978	0.574	0.905 – 1.057
	**Yorkshire and the Humber**	1.364	<0.001	1.255 – 1.482
	**East Midlands**	1.165	0.001	1.068 – 1.271
	**West Midlands**	1.316	<0.001	1.212 – 1.428
	**East of England**	0.897	0.013	0.823 – 0.977
	**London**	1.017	0.700	0.934 – 1.108
	**South East Coast**	0.832	<0.001	0.761 – 0.909
	**South Central**	0.783	<0.001	0.713 – 0.861
	**South West**	0.965	0.409	0.886 – 1.051

* Inclusion of the access variable in the model resulted in a statistically significant improvement in model fit; likelihood ratio test statistic  = 18.78; *P*<0.001.

The median rate of self-referred discharged ED visits for practices in the first quintile was 0.098 (IQR: 0.063–0.136); if this rate was 10.2% lower for a practice with a median registered population size (for the first quintile, 6,464 registered patients), 65 fewer visits per year are expected. If the rate was 10.2% lower for all practices in the first quintile (n = 1,576), the model predicts 111,739 fewer self-referred discharged ED visits per year across the entire NHS. The cost to the NHS of a visit at an ED is £54 ($82; €63) or above [17]; a conservative estimate for the cost saved from 111,739 fewer ED visits is therefore £6,033,906 ($9,208,344; €7,056,201).

Several of the control variables also had a statistically significant association with the outcome variable. A one unit increase in the percentage of the registered population that had tried to see a GP within two weekdays predicts a 0.7% (RR = 1.007; *P*<0.001) increase in the rate of self-referred discharged ED visits. In contrast, a one unit increase in the average travel time to the nearest hospital relative to that to the nearest GP by public transport and/or walking predicts a 2.6% (RR = 0.974; *P*<0.001) decrease in the visit rate. The two variables with the largest effects are the urban/rural classification of a general practice’s location and the IMD score for its registered population. Practices located in rural areas had a 15.0% (RR = 0.850; *P*<0.001) lower rate of ED visits than those in urban areas. Relative to practices with registered populations in the least deprived quintile, those with populations in the most deprived quintile had a 41.7% (RR = 1.417; *P*<0.001) greater rate of visits. The percentage of the registered population aged 65 years or over had a statistically significant negative association with the outcome variable; a one unit increase in this percentage predicts a 1.1% (RR = 0.989; *P*<0.001) decrease in the rate of ED visits. Further, the prevalence of obesity in the registered population had a statistically significant positive association with the rate of ED visits (RR = 1.006; *P* = 0.021), whereas the prevalence of asthma and hypertension did not. Finally, the Strategic Health Authority in which a practice is located also explained some of the variation in the outcome variable; for example, the model predicts a 36.4% (RR = 1.364; *P*<0.001) greater rate of visits for practices in Yorkshire and the Humber, relative to those in North East.

## Discussion

The percentage of the registered population that was able to see a GP within two weekdays, a measure of timely access to primary care, was negatively associated with the rate of self-referred discharged ED visits. Our findings support the hypothesis that some patients who are unable to see a GP within two weekdays self-refer to an ED [3] and are subsequently discharged. In 2011-12, 9% of respondents to the GP Patient Survey who were unable to obtain a convenient appointment on their last attempt report subsequently going to an ED or walk-in centre [18], which accords with the results of our analysis.

ED visits were also associated with a number of other variables, including age, socioeconomic status, and urban/rural location, indicating that such visits may reflect differences in health beliefs, health-seeking behaviour and doctor-patient relationships between groups [19]. However, even in a multivariable analysis that adjusted for all these factors, timely access to general practice services remained a significant predictor of self-referred discharged ED visits. The measure of GP supply did not have a statistically significant association with the outcome variable in the multivariable analysis. We infer that this characteristic of the health system does not influence ED visits independent of its effect on timely access to primary care.

Previous research of 68 general practices in London, England did not identify a statistically significant association between patient-reported access to general practice services and the rate of self-referred discharged ED visits [5], possibly due to insufficient statistical power. This explanation may also apply to a similar analysis of 145 practices in Leicestershire, England, which included all types of ED visit in the outcome variable [6]. A relative strength of the analysis presented here is the greater number of general practices included and their distribution throughout England, providing greater power to detect true associations and results that are directly generalisable nationally. Its findings complement a series of studies that report a negative association between patients’ ability to obtain a GP appointment and emergency admissions for several primary care sensitive conditions in England, including cancer, chronic obstructive pulmonary disease and stroke [20]–[22]. The positive association between the IMD score for a general practice’s registered population and the outcome variable is consistent with the findings of previous studies in England [5], [6], [23]–[25].

In the U.S., the use of EDs by Medicaid patients is lower for those enrolled in primary care practices providing extended after-hours care or practices with fewer active patients per clinician-hour [26], and patient-reported barriers to timely access to a usual source of medical care are associated with ED use [27]. Research from Canada has shown that access to a primary care physician is a significant predictor of ED use in low severity cases [28] and a low continuity of care or no access to a primary care physician is associated with an increased rate of ED use [29]. These findings are consistent with those presented here.

The study has addressed a topical, policy-relevant research question using national data for England. The analysis included a range of variables from established datasets, providing original evidence for the relation between access to primary care and ED visit rates, whilst controlling for several variables.

Yet, due to the cross-sectional, population-based design of the analysis, the observed associations may not be inferred for individual patients and the temporal nature of these associations cannot be ascertained. However, some individuals do attend an ED after being unable to obtain a GP appointment [3], [18], making a causal relationship at the patient level plausible. Some providers did not submit data to Hospital Episode Statistics in 2010-11, such that the ED data used had records for 94% of visits that occurred during the year [30]. The GP Patient Survey had a median response rate of 40% (IQR: 32–47%); practices’ scores for the percentage of the registered population that was able to see a GP within two weekdays are not associated with response rates [31] and so the potential for selection bias is limited. The definition of the travel time variable assumes that the nearest hospital has an ED and that a patient will attend, via public transport and/or walking, the nearest service. Although this may not apply in all cases, it was important to estimate the relative travel time due to its importance as a determinant of service utilisation [6], [32]–[34].

The practice level findings presented here require confirmation with a similar analysis conducted at the patient level. By collecting the same data for future years, a longitudinal analysis could test whether improvements in access to primary care over time reduce the rate of ED visits. This analysis could also examine whether the current financial pressures facing general practices in England result in poorer access to primary care and an increase in the rate of ED visits [35]. A cluster-randomised controlled trial of practices with different access arrangements would provide more definitive evidence to support or challenge the presented hypothesis.

The analysis supports the hypothesis that enabling patients to see a GP in a timely manner could reduce ED utilisation in the NHS in England, a health system providing universal coverage for primary care. In countries where some patients experience financial barriers to accessing primary care, such as in the U.S. [2], the association could be more profound. The economic crisis in Europe has caused some governments to adopt policies that increase financial barriers to primary care, which could lead to increased utilisation of EDs and hospital care more generally [36]. Yet, the findings of this study indicate that even in a system with universal coverage, barriers to primary care access persist and are sufficient to influence ED utilisation. What remains in England is how the current extensive NHS reforms [37] might impact on such an association.
